# Items

**Published:** 1902-02

**Authors:** 


### ITEMS.

THE attention of the profession of medicine is invited to the advertisement of the Roberts Lymph Sanitarium, on page xxiv. Dr. Edward T. Smith who conducts it is one of our very reputable physicians, and his institution is vouched for by other prominent medical men.

THE Columbia calendar for 1902, is a useful memorandum and desk reminder, not only of the days of the week and month, but of Columbia and Hartford bicycles that are now so popular. The Columbia bicycle now enters upon its 25th year, and the calendar upon its 17th year—both able to stand on their own merits, and are made by the American Bicycle Company, Hartford, Conn.

THE Scott and Bowne desk and memorandum calendar for 1902 has presented itself in its usual good form. It has become a regular and well-nigh indispensable visitor, for no desk is complete without some such ready reminder of duties that otherwise might be forgotten. This one, too, is a reminder of the many uses, new and old, of Scott's emulsion that must prove of value to a vast number of physicians. It will be sent on application to any who may have been overlooked in its distribution.

THE Antikamnia calendar for 1902 presents a reproduction in colors of Helen Hyde's "Her first picture book." It is an attractive copy of the original painting which Mr. Ruf, the president of the company, purchased last year in Paris. On the reverse is the calendar proper together with a few remarks relating to the products of the Antikamnia Chemical Company, St. Louis.

MISS MARY I. CAMPBELL announces that the partnership between herself and Mrs. Amy G. Goodwyn has been dissolved, and that the private sanitarium at No. 50 Allen Street, Buffalo, is now entirely under the direction of Miss Campbell.

Miss Bertha Baldwin, of Brantford, Ont., a graduate of the Long Island College Training School, has been engaged as resident nurse. Miss Baldwin graduated with high honor in 1900 and is eminently qualified for her present position.

Although the sanitarium is principally for the care of out of town patients, residents of Buffalo will also be received when there is room. A room has been especially fitted up for treatment, such as vapor baths, massage, and other similar purposes.

The Varoma Medical Company has succeeded to the business of the Vaporia Medical Co., and the name "Varoma" has been substituted for "Vaporia."

Schieffelin & Co., New York, are acting as sole agents for the Varoma Medical Company, and all orders and inquiries may be addressed to them.

Messrs. Schieffelin make the following statement in regard to the product:

Varoma is a combination of various volatile coal tar derivatives, possessing high antiseptic powers, and yet perfectly innocuous. The vapor of varoma is unirritating, free from unpleasant odor, and may be safely breathed by the youngest child. In cases of laryngeal diphtheria (croup) and whooping cough, if the air of the sickroom is constantly charged with the vapor, the disease will be found to run a much milder course. At the same time the spread of the disease to other children or the members of the household will be prevented.

Aside from its use in the above class of patients, varoma may be employed with benefit in other affections of the respiratory organs. In tuberculosis of the lungs, it promises to be of great service by exerting a local antiseptic influence upon the affected lung tissues and also destroying the bacilli in the sputum and in the expired air. Excellent results have also been obtained with varoma in cases of pneumonia, bronchitis, asthma and hay fever, its effect here being to allay irritation, subdue spasm, and render expectoration easier.

As a general disinfectant agent for purifying the air of the house during and after the occurrence of contagious diseases, such as measles, scarlet fever, typhoid, smallpox, etc., varoma will be found to possess decided advantages over the non-volatile antiseptics, since the vapor penetrates to parts that cannot be reached by fluids. The fact that it will not injure clothing, furniture, paintings, draperies or delicate fabrics renders it most eligible for this purpose.

The vaporiser furnished with varoma is inexpensive, convenient for application and embodies some entirely new features, rendering it the most perfect in use.

---

A Century of Medical History.—The history of medicine in Erie County for the nineteenth century, which has been running through many numbers of the Journal, is drawing towards completion. Within a short time a limited number of copies will be offered to those who may desire them at $1.00 net a volume. It will make a book of over 150 pages, profusely illustrated and containing material that is of interest to every physician in the county. In order that we may be advised as to the size of the edition to print it is important that orders be placed at once with

William Warren Potter, M. D.,
284 Franklin Street, Buffalo, N.Y.

